# Effect of tDCS with an extracephalic reference electrode on cardio-respiratory and autonomic functions

**DOI:** 10.1186/1471-2202-11-38

**Published:** 2010-03-16

**Authors:** Yves Vandermeeren, Jacques Jamart, Michel Ossemann

**Affiliations:** 1 Neurology Department, Cliniques Universitaires UCL de Mont-Godinne, Université catholique de Louvain (UCL), Avenue Dr G. Therasse, Yvoir 5530, Belgium; 2 Université catholique de Louvain (UCL), Institute of NeuroScience (IoNS), Avenue Hippocrate, 54 Bte 54.10, Brussels, B-1200, Belgium; 3 Scientific Support Unit, Cliniques Universitaires UCL de Mont-Godinne, Université catholique de Louvain (UCL), Avenue Dr G. Therasse, Yvoir 5530, Belgium

## Abstract

**Background:**

Transcranial direct current stimulation (tDCS) is used in human physiological studies and for therapeutic trials in patients with abnormalities of cortical excitability. Its safety profile places tDCS in the pole-position for translating in real-world therapeutic application. However, an episode of transient respiratory depression in a subject receiving tDCS with an extracephalic electrode led to the suggestion that such an electrode montage could modulate the brainstem autonomic centres.

We investigated whether tDCS applied over the midline frontal cortex in 30 healthy volunteers (sham n = 10, cathodal n = 10, anodal n = 10) with an extracephalic reference electrode would modulate brainstem activity as reflected by the monitoring and stringent analysis of vital parameters: heart rate (variability), respiratory rate, blood pressure and sympatho-vagal balance.

We reasoned that this study could lead to two opposite but equally interesting outcomes: 1) If tDCS with an extracephalic electrode modulated vital parameters, it could be used as a new tool to explore the autonomic nervous system and, even, to modulate its activity for therapeutic purposes. 2) On the opposite, if applying tDCS with an extracephalic electrode had no effect, it could thus be used safely in healthy human subjects. This outcome would significantly impact the field of non-invasive brain stimulation with tDCS. Indeed, on the one hand, using an extracephalic electrode as a genuine neutral reference (as opposed to the classical "bi-cephalic" tDCS montages which deliver bi-polar stimulation of the brain) would help to comfort the conclusions of several modern studies regarding the spatial location and polarity of tDCS. On the other hand, using an extracephalic reference electrode may impact differently on a given cortical target due to the change of direct current flow direction; this may enlarge the potential interventions with tDCS.

**Results:**

Whereas the respiratory frequency decreased mildly over time and the blood pressure increased steadily, there was no differential impact of real (anodal or cathodal) *versus *sham tDCS. The heart rate remained stable during the monitoring period. The parameters reflecting the sympathovagal balance suggested a progressive shift over time favouring the sympathetic tone, again without differential impact of real *versus *sham tDCS.

**Conclusions:**

Applying tDCS with an extracephalic reference electrode in healthy volunteers did not significantly modulate the activity of the brainstem autonomic centres. Therefore, using an extracephalic reference electrode for tDCS appears safe in healthy volunteers, at least under similar experimental conditions.

## Background

Repetitive transcranial magnetic stimulation (rTMS) and transcranial direct current stimulation (tDCS) can modulate brain excitability and behaviour in healthy volunteers [1,2]; numerous proof-of-principle studies have demonstrated their therapeutic potential in patients with disease resulting from or leading to abnormalities of brain excitability such as Parkinson's disease, stroke, tinnitus, chronic pain and depression [3-5]. Under different forms and names, brain polarisation has been applied extensively in human subjects since centuries (for an extensive review, see ([6]). It is generally admitted that the effects of tDCS are less focal than those of rTMS and despite the development of realistic head models uncertainties remain about the distribution of the direct current (DC) within the brain. This issue is crucial since it has been suggested that the current flow direction relative to the neuronal elements may be one of the key factors driving the effects of tDCS [7-13]. Interestingly, an episode of transient respiratory depression in a healthy volunteer under frontal tDCS with an extracephalic reference electrode suggested that this electrode montage could lead to a modulation of the brainstem respiratory centres [14,15]. When Nitsche and Paulus meticulously expanded the seminal findings of the impact of tDCS on the primary motor cortex (M1) excitability described by Priori and collaborators [16-18], they cautiously banned the use of an extracephalic electrode and proposed the now "classical" montage for modulating M1 excitability with tDCS: the "active" electrode over the target M1 and the other "reference" electrode over the contralateral orbita [19].

Recently, some investigators applied tDCS with an extracephalic reference electrode, without reporting adverse events [20-24]. Accornero and collaborators concluded that the use of an inion-neck montage did not carry an extra risk since heart rate, blood pressure and body temperature remained unchanged during tDCS and 20 minutes after in healthy volunteers [20]. However, these conclusions were limited since the respiratory frequency was not monitored and since the effects on the autonomic nervous system were not explored.

Therefore, in order to explore whether tDCS with an extracephalic reference electrode would modulate the autonomic functions of the brainstem, respiratory frequency, blood pressure and heart rate were continuously monitored before, during and after tDCS in 30 healthy volunteers. The vital parameters and parameters reflecting the activity of the autonomic nervous system were extracted and compared between the anodal, cathodal and sham tDCS groups.

We reasoned that this study could lead to two opposite but equally interesting outcomes:

1) Applying tDCS with an extracephalic electrode could induce modulations of the vital parameters and/or in the sympatho-vagal balance. Whereas the stimulation of the brainstem by DC may appear at first sight as an unwanted and potentially dangerous side effect, this might on the other hand result in the development of an attractive new tool to explore non-invasively the autonomic nervous system in human subjects. The modulation of the activity of the brainstem autonomic centres by tDCS may even open new therapeutic perspectives. Indeed, the crucial role of the autonomic nervous system in the regulation of the major homeostatic functions has long been known [25,26]. Alternatively, targeting the cortical areas involved in the control of the brainstem autonomic centres may also be envisaged as a therapeutic intervention as suggested recently [18]. Whether an indirect modulation of the brainstem autonomic centres through cortical areas would be more effective than a direct modulation of the brainstem by tDCS remains speculative.

2) On the opposite, if applying tDCS with an extracephalic electrode had no effect on these parameters, it could thus be used safely in healthy human subjects. This outcome would significantly impact the field of non-invasive brain stimulation with tDCS. Indeed, the use of an extracephalic electrode as a genuine neutral reference (as opposed to the bi-cephalic montage which deliver bi-polar stimulation of the brain) would help to confirm the conclusions of several modern tDCS studies using the "bi-cephalic" tDCS montages regarding the importance of DC flow direction, polarity, spatial location, and the simultaneous stimulation of two brain regions by opposite polarities. Whereas the measures of the cortical excitability of M1 after tDCS are not influenced by a "reference" electrode placed over the controlateral frontopolar cortex, the issue is much more debatable for cognitive studies and studies using behavioural outcome measures (reaction time, force, decision, etc.).

## Methods

This exploratory study assumed a single-blind, sham-controlled, parallel-group design. The 30 healthy volunteers were randomly assigned to receive a single 20-minutes session of anodal (n = 10), cathodal (n = 10) or sham (n = 10) tDCS; they were tDCS-naïve and blinded to the nature of the tDCS intervention.

### Subjects

The protocol was approved by the local Ethical Committee (Comité d'éthique médicale des Cliniques universitaires UCL de Mont-Godinne, Yvoir, Belgium) and the study was conducted according to the recommendations of the Helsinki declaration. The 30 healthy volunteers provided written informed consent at inclusion. The inclusion criteria were 1) being a healthy volunteer with no known disease, 2) being between 20 and 60 years of age. The exclusion criteria were 1) pregnancy, 2) presence of any chronic disease, 3) intake of drugs modulating vital parameters (beta blockers, blood pressure lowering drugs) or with a central action (antihistaminic, antidepressant, antiepileptic), 4) presence of a pacemaker or any intracranial metal. The three groups were balanced for age and sex; demographic and characteristics baseline heart rate, blood pressure are listed in Additional file 1. The subjects were instructed to avoid consuming caffeine, tea, alcohol or psycho-active drugs since the day before the experiment. One female subject in the cathodal group (# 14) was a regular smoker, she avoided smoking overnight.

### tDCS

Since the aim of the study was to detect whether tDCS applied with an extracephalic electrode would modulate the cardio-respiratory or autonomic functions at the level of the brainstem, we used a montage comparable to that used by Lippold and Redfearn [14,15], in order to maximise the current flow through the brainstem, except that a single electrode was applied over the scalp. The active electrode (35 cm^2^) was placed on the midline over Fz and the extracephalic reference electrode (35 cm^2^) was placed over the right tibia. tDCS electrodes were soaked with a standard saline solution (NaCl 9%) and maintained by elastic bands. An Eldith^® ^DC-stimulator (neuroConn GmbH, Ilmenau, Germany) delivered 20 minutes of anodal or cathodal stimulation (1 mA, fade in/out 8 s). For sham stimulation, 30 s of stimulation were applied (1 mA, fade in/out 8 s, 14 s on); the polarity of the frontal electrode for the sham tDCS was anodal in half of the healthy volunteers.

Before starting the 1-hour monitoring period, each healthy volunteer was subjected to approximately 20 s of tDCS (1 mA, fade in 8 s, 12 s on, manual termination by the experimenter), in order to attenuate anxiety and the novelty effect during the subsequent monitoring period. Such stimulation is known to have only very transient effect on cortical excitability [19]. By giving this short tDCS stimulation, the subject were familiarised with the cutaneous itching sensation before the monitoring period and reassured about the benignity of tDCS, thus minimising changes in heart rate, blood pressure and respiratory frequency linked to anxiety.

After this short tDCS familiarisation, the monitoring electrodes were placed, the experiment was re-explained to the subject, calibrations and adjustments were performed. Altogether, at least ten minutes elapsed between the end of the brief tDCS familiarisation and the onset of the 1-hour continuous monitoring period.

### Monitoring

The 1-hour monitoring period was divided in three successive epochs: baseline (20 minutes), intervention (20 minutes) and post-intervention (20 minutes). Monitoring was performed in a quiet room with a dim light; the room's temperature was kept constant throughout the monitoring period (22°C). After lying supine in a bed, the subjects received the instruction to relax as much as possible without falling asleep, to keep a regular self-paced respiratory frequency and to avoid talking. They were kept awake by the investigators who continuously talked to them and checked their wakefulness state, as well as the appearance of any discomfort. In some occasions, the subjects were allowed to talk briefly in order to avoid falling asleep. On-line monitoring of the vital parameters was performed by visual inspection of the displayed data and trends.

Respiratory frequency (RF, Hz) monitoring was performed through a piezo-electric sensor (Sleepmate^®^, Ambu Inc, Maryland, United States) mounted on an adjustable elastic belt positioned over the xiphoid process. Digital markers were used to define the start of tDCS, end of tDCS, and any events during the monitoring period. The respiratory waveform was recorded with a conventional multichannel EEG system (Brainnet^® ^MEDATEC, Brussels, Belgium), filtered (0.18 Hz - 16 Hz), with a 200 mV gain. During off-line analysis, the respiratory frequency (Hz) was manually edited over periods of 30 seconds which were then concatenated in 5 minutes bins for statistical analysis.

Continuous monitoring of blood pressure and heart rate was performed with a Task Force^® ^Monitor 3040i (CNSystems Medizintechnik GmbH, Graz, Austria). Blood pressure (mmHg) was continuously monitored at the index or third finger of the right hand with a special finger cuff, alternating between the index and third finger cuff when subject mentioned discomfort (verbally checked every five minutes). A control oscillometric measure of the blood pressure (mmHg) was performed every five minutes through a standard blood pressure cuff placed on the left arm. The Task Force Monitor V2.1 software allows the continuous monitoring and recording of beat-to-beat systolic (sBP) and diastolic (dBP) blood pressure.

Continuous heart rate (HR) monitoring in beat per minute (bpm) was performed through four surface electrodes placed on the deltoid muscles and over the twelfth ribs along the median axillar line on both sides. The ground electrode was placed on the left leg. The Task Force^® ^Monitor was synchronised with the BrainNet^® ^respiratory monitoring system; digital markers (onset of tDCS, end of tDCS) were simultaneously added to both systems to ensure synchronicity.

For offline analysis, the following parameters were extracted for statistical analysis: the heart rate (HR, in bpm), the systolic (sBP) and diastolic (dBP) blood pressures (mmHg). In order to evaluate the sympathetic and parasympathetic tones, power spectra analysis of the R-R interval (RRI) were brought into the frequency domain by the adaptive autoregressive parameter algorithm implemented in the Task Force Monitor V2.1 software. The sympathetic and vagal tones of the human autonomic nervous system can be estimated from power spectra analysis of heart rate variability (HRV) [27-32]. The sympathetic tone is reflected by the low frequency band (LF-band: 0.04-0.15 Hz) of the HRV, whereas the vagal tone is reflected by the high frequency band (HF-band: 0.15-0.4 Hz) partly contaminated by the RF (respiratory sinus arrhythmia, RSA) [28,32]. The following parameters were computed and extracted for off-line statistical analysis: LF nu-RRI (sympathetic tone of RRI in normalised unit (nu), %), HF nu-RRI (vagal tone of RRI in normalised unit (nu), %), LF/HF-RRI (sympathovagal balance of RRI), and PSD-RRI (Power Spectral Density of RRI, ms^2^). The number of ectopic beats by epoch was counted separately for each subject.

### Statistical analysis

Statistical analyses were performed using the SPSS^® ^15.0 statistical software (SPSS Inc., Chicago, Ill). First, in order to disclose gross changes between epochs and types of stimulation, a repeated measures analysis of variance (ANOVA_RM_) was performed by the multivariate procedure and T^2 ^Hotelling test to compare the mean and SD values of each group and each epoch (baseline, intervention, post-intervention).

Then, the mean and SD values of each parameter were concatenated in bins of 5 minutes and compared in order to detect potential changes within and between the epochs. These 5 minutes bins of the mean and SD values were analyzed by regression of repeated measures using generalized estimating equations (GEE) to take into account the multiplicity of inter-correlated values in each subject [33]. GEE models used a normal probability distribution, an identity link function and an exchangeable working correlation matrix structure. A first GEE model compared the baseline epochs of the 3 groups to rule out baselines differences between groups, which would prompt to compute GEE analysis separately for each group. A second GEE model used sex, age, group, epoch and an interaction term epoch-group as covariates with respect to the baseline epoch considered as common for the 3 groups after the use of the first model. Since this study was designed for exploring the potential of tDCS with an extracephalic reference electrode to modulate the activity of the brainstem autonomic centres, we intentionally decided to avoid using corrections for multiples comparisons such as Bonferroni correction.

Finally, the percentages of parameters values outside the limits defined by the mean ± 1.96 SD computed on the baseline values of each subject were also considered. They were compared between groups by Kruskal-Wallis analysis of variance by ranks for each parameter and each epoch, and between phases by Friedman test for each parameter and each group.

## Results

Apart from transient itching sensation under the tDCS electrodes in several subjects, the unique side-effect reported was the occasional perception of phosphenes during the ramp in of tDCS (n = 2). Whereas phosphenes have never been reported using a ramping up current, defective electrode contact is ruled out by the fact that no error message was provided by the Eldith DC stimulator^®^. We can only speculate that this electrode montage may be particularly prone to induce phosphenes. It is also worth noting that the occurrence of phosphenes was explicitly mentioned before familiarisation as a potential side effect in order to avoid excessive stress reaction; some subjects may have been particularly receptive to suggestion or attentive to visual effects. None of the subject reported discomfort, breathing difficulty or palpitation; there was no drop-out. During the experiment, no change was observed by on-line monitoring for any of the parameters of interest.

**Figure 1:**
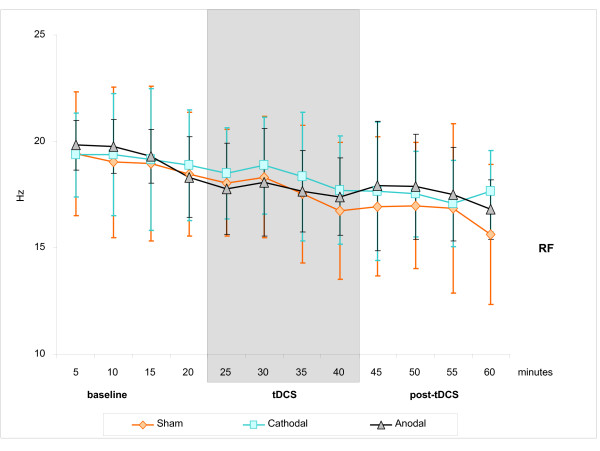
**Temporal evolution of the RF for each group (sham, cathodal, anodal)**. Mean +/- 1 SD of the RF by bins of 5 minutes over the monitoring period (3 epochs: baseline, tDCS, post-tDCS).

**Figure 2:**
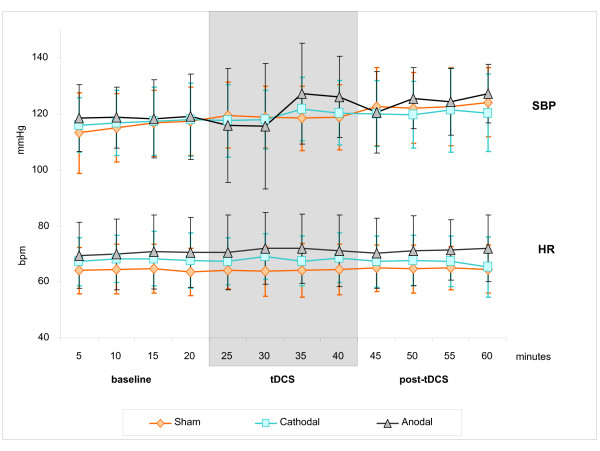
**Temporal evolution of the sBP and HR for each group (sham, cathodal, anodal)**. Mean +/- 1 SD of the RF by bins of 5 minutes over the monitoring period (3 epochs: baseline, tDCS, post-tDCS).

Using one value per 20-minutes epoch (baseline, tDCS, post-tDCS) per subject, the ANOVA_RM _failed to disclose any statistically significant overall difference for the means and SDs of any parameters between groups. However, the ANOVA_RM _showed a significant effect of epochs, with a decrease in the RF (p < 0.001, see Figure 1), not significantly different between groups (p = 0.805). Similarly, there was an increase in the mean sBP (p < 0.001) and dBP (p = 0.001), as depicted in Figure 2. Whereas the mean and SD HR remained stable (p = 0.960 and 0.854, respectively; see Figure 2), there was an increase in the mean LF nu-RRI and a mirroring decrease in the mean HF nu-RRI (p = 0.001); a consequent increase in the mean PSD-RRI (p = 0.031) and in the LF/HF ratio (p = 0.022). There was neither a statistically significant difference between groups (all p > 0.137) nor any significant group by epoch interaction (all p > 0.141). Hence, whereas the parameters changed gradually over time, these changes were similar between groups.

As estimated by the first GEE model, baseline data for the three groups were not statistically different when comparing the mean and SD values of the following parameters: RF, HR, sBP, dBP, PSD-RRI and the LF/HF-RRI ratio (all p values > 0.143). Whereas the mean LF nu-RRI and HF nu-RRI baselines were not statistically different (p > 0,215), the SD values for LF nu-RRI (HF nu-RRI) of the sham group were significantly smaller than in the anodal (p = 0.020) and lower than in the cathodal (p = 0.068) groups, which prompted to compute separate GEE models for each group.

Then, age, sex, group, epoch and interaction epoch-group were used as covariates in the second GEE regression in order to search for more subtle changes between the mean and SD values of the parameters of interest. In accordance with the global ANOVA_RM_, the second GEE model showed that the RF decreased significantly and steadily throughout the monitoring period (p = 0.021), without significant difference between groups (p = 0.561) nor group by epoch interaction (p = 0.459). Sex also influenced the RF since the RF was lower in male than in female subjects (p = 0.033).

The mean sBP and dBP increased significantly over time (p = 0.010 and 0.001, respectively, see Figure 1); this continuous rise of the blood pressure over the monitoring period was not different between groups (p > 0.28) and there was no significant group by epoch interaction (p > 0.55). Age and sex significantly impacted blood pressure: the mean sBP was higher in younger than in older subjects (p = 0.006) and also higher in male than in female subjects (p < 0.001); the SD of the sBP was larger in older than in younger subjects (p = 0.048). The mean dBP was higher in male than in female subjects (p < 0.001), whereas the SD of the dBP was larger in female than in male subjects (p = 0.044).

The mean and SD of the HR did not change significantly over time and seemed not influenced by group, age or sex (all p > 0.093, Figure 1). Whereas ANOVA_RM _demonstrated a significant global effect of epoch on the LF nu-RRI and HF nu-RRI, the GEE model showed only a non-significant trend for an increase of the mean LF nu-RRI and a mirror decrease of the mean HF nu-RRI (p = 0.056) over the monitoring period. In addition, the GEE model also disclosed a significant overall effect of group (sham group with a lower mean LF nu-RRI and a higher mean HF nu-RRI than the anodal and cathodal groups, p = 0.026). However, there was no significant group by epoch interaction (p = 0.584). Since the baseline SD values of the LF nu-RRI and HF nu-RRI were statistically different, separate GEE models were computed for each group; these separate GEE models showed no significant effect for the sham and anodal groups (p > 0.277) but showed a significant decrease (increase) of the SD values for the LF nu-RRI (HF nu-RRI) over time for the cathodal group only (p = 0.044). Again, whereas ANOVA_RM _seemed to demonstrate a significant global effect of epoch suggesting an increase of the PSD-RRI and LF/HF ratio, the GEE model failed to find any significant effect of phase, group, age, sex nor group by epoch interaction (all p > 0.101) for the mean and SD values of the PSD-RRI and LF/HF ratio.

Infrequent ectopic beats were observed in only two healthy volunteers (# 9 and #19); these ectopic beats were randomly distributed throughout the epochs.

Finally, the percentages of values outside the mean ± 1.96 SD did not differ significantly between the three groups for any parameter and any epoch. There was a trend for an increase of these percentages between epochs 1, 2 and 3, which was significant for sBP and dBP in the three groups (p = 0.003 and p = 0.014 respectively), for PSD-RRI in the cathodal group (p = 0.020) and for LF/HF-RRI in the cathodal (p = 0.027) and anodal (p = 0.007) groups.

## Discussion

Thirty healthy volunteers were subjected to 1 mA tDCS during 20 minutes with a mid-line frontal electrode (anodal n = 10, cathodal n = 10, sham n = 10) and an extracephalic reference electrode, under continuous cardio-respiratory monitoring. No adverse effect was observed. While the RF decreased progressively, the blood pressure increased steadily over time, without significant difference between groups. The HR remained stable during the monitoring period. The HRV parameters reflecting the tones of the sympathetic and vagal autonomic nervous system suggested a progressive shift in the sympathovagal balance favouring the sympathetic tone. Neither anodal nor cathodal tDCS modified the sympathovagal balance when compared to sham tDCS.

### tDCS and the brainstem

Lippold and collaborators reported an episode of disturbed speech and apnoea followed by a transient respiratory depression in a normal female subject who received 16 minutes of 3 mA bi-frontal cathodal tDCS, with an extracephalic reference electrode [14,15]. The authors concluded that the respiratory depression was due to an unwanted modulation of the brainstem respiratory centres by the DC flowing through the brainstem [14,15]. Recently, tDCS with an extracephalic reference electrode has been safely applied using various montages: inion and neck base [20], M1 and ipsilateral shoulder ([21] and in two patients with Tourette syndrome [22], left fronto-temporal areas or inion and right shoulder [22], bi-frontal and non-dominant arm [23], bilateral dorsolateral prefrontal cortex and right deltoid [34], cerebellum [35]. However, monitoring of the respiratory frequency or exploration of subtle changes in the sympathovagal balance have not been conducted so far, leaving unanswered the issue whether tDCS could modulate the activity of the brainstem autonomic nervous nuclei.

If tDCS with an extracephalic reference electrode could modulate the activity of the brainstem cardio-respiratory and autonomic centres, this would lead to the exciting possibility to directly test and manipulate homeostatic functions such as respiratory frequency, heart rate. Moreover, this could lead to therapeutic perspectives since the autonomic nervous system is an essential target for pharmacological therapies given its key role in hypertension, heart failure, cardiac arrhythmias, sudden death or dysrhythmic breathing [36-39].

### Respiratory frequency

In the present experiment, the extracephalic reference electrode was placed on the right leg and a unique active tDCS electrode over Fz, to maximise the likelihood of orienting the DC flow through the brainstem. The RF decreased steadily throughout the 1-hour monitoring. This mild diminution of the RF could be explained by a progressive relaxation while lying supine. When necessary, the healthy volunteers were allowed to speak briefly to stay awake, mostly during the last 30 minutes of monitoring. Although this may partly explain the decrease of the measured RF in the three groups, this trend was present since the onset of the monitoring period. Nevertheless, there was no significant difference in the temporal evolution of the RF between the sham, anodal and cathodal groups. Therefore, 20-minutes of tDCS applied with an extracephalic reference electrode seem not to interfere with the activity of the brainstem respiratory network and thus appear to be safe in healthy volunteers. Of course, longer stimulation periods, higher tDCS intensities, other electrode montages and the inclusion of patients should be performed before this conclusion can be generalised.

### Blood pressure

The blood pressure increased mildly throughout the monitoring period from the onset of the experiment, suggesting an increasing nervousness during the monitoring period rather than transient anxiety at the onset of intervention. This hypothesis is supported by the fact that the HR remained stable over time (see below). The blood pressure remained within the normal range for most of the healthy volunteers during the baseline epoch; some of them showed values compatible with previously unknown mild hypertension, mostly by the end of the monitoring period. The variability outside the mean ± 1.96 SD also increased throughout the epochs in all three groups. Anyway, the blood pressure followed a similar temporal evolution in the three groups.

### Heart rate and sympathovagal balance

The HR remained stable over time in the three groups, ruling out transient stress at the onset of intervention or a significant effect of tDCS on HR. There was a significant increase of the LF nu-RRI and a mirroring decrease of the HF nu-RRI over time, as well as resulting increases in the LF/HF ratio and PSD-RRI. These progressive changes were similar for the three groups, suggesting a progressive shift of the sympathovagal balance in favour of the sympathetic tone.

Could this shift of in the power of the LF and HF bands have been driven by the decline of RF? Indeed, whereas the HF band of HRV reflects the vagal tone, it is also partly contaminated by the RF, a phenomenon called the respiratory sinus arrhythmia (RSA) [28,32]. Tasks such as mental arithmetic test or free talking may shift respiration and the HRV balance within the LF band [40]. In the present experiment, whereas the RF decreased mildly over time, the LF nu-RRI increased. However, since the R-R variability is almost abolished after autonomic ganglion blockade of both the sympathetic and parasympathetic nervous systems in healthy volunteers during supine resting condition, it has been suggested that the HRV is predominantly mediated by the autonomic nervous system [31]. Overall, there was a progressive shift of the sympathovagal balance in favour of the sympathetic tone, similar in the three groups, likely reflecting an increasing nervousness. It is of course acknowledged that the concept of sympathovagal balance is a coarse approximation and does not fully reflect the complex interactions of the sympathetic and vagal systems [28,32,41]

In addition, the GEE model disclosed subtle differences that were not picked up by the ANOVA_RM_. The LF nu-RRI was lower (and the HF nu-RRI higher) in the sham group than in both the anodal or cathodal group but, again, there was no epoch by group interaction. Similarly, the percentage of data outside the mean ± 1.96 SD increased for the LF/HF-RRI ratio in the anodal and cathodal groups. In order to explain this difference between the sham group on the one hand and the anodal and cathodal groups on the other hand, one should hypothesize that both anodal and cathodal tDCS induced the same changes in the sympathovagal balance whereas sham intervention had no influence. This would imply that the DC "interfered" with the activity of these brainstem centres independently of its flow direction, resulting in the same net effect. Alternatively, whereas subjects undergoing tDCS are unable to explicitly point out whether the tDCS stimulation is on (active) or off (sham) [42], they still may unconsciously perceive the active stimulation and experience subliminal stress. This would hold true only if this change started after the onset of intervention; however it was already present during the baseline period and built-up steadily.

The separate GEE models disclosed a decrease in both the LF nu-RRI and HF nu-RRI variability exclusively in the cathodal group; this was reflected in the fact that the analysis of the percentage of variance outside the mean ± 1.96 SD also showed a difference for the PSD-RRI for the cathodal group only. This isolated result is difficult to interpret since the baseline difference that prompted to compute separate GEE models concerned the sham group versus the anodal and cathodal group.

### Asymmetry of the DC flow

Could the 1964 episode of respiratory depression [14,15] result from an asymmetrical distribution of the DC within a specific part of the brainstem or on its right aspect (the extracephalic electrode was on the right leg)? Lateralised tDCS with an extracephalic electrode could theoretically impact on cardio-respiratory homeostasis through three additional mechanisms. First, when applied over the lateral aspect of the head, the DC could potentially modulate the activity of the cortical areas involved in the control of autonomic nervous functions such as the insula and amygdala [43,44]. Whereas the debate about the hemispheric lateralisation of autonomic control in human is still open [45], the insula and parietal cortices may be particularly important in the control of heart rate and have been involved in sudden cardiac death and cardiac arrhythmia after stroke [39,46,47].

Second, since DC can modulate the excitability of peripheral nerves [48], the DC flowing preferentially through the lateral aspect of the neck could theoretically interfere with the vagus nerve excitability. It is worth noting that the right vagus nerve seems conveying a predominant outflow toward the heart [49]. Recently, two out of three patients receiving right vagus nerve stimulation for refractory epilepsy suffered from respiratory events suggestive of bronchoconstriction [50]. Therefore, it cannot be ruled out that episode of respiratory depression under tDCS with a right extracephalic electrode [14,15] was due to a modulation of the right vagus nerve excitability, resulting in breathing difficulties.

Third, the phrenic nerve might also be influenced by DC, maybe leading to a modulation of the RF and respiratory depression. However, whereas unilateral paralysis of the phrenic nerve may result in hemidiaphragmatic paralysis leading to severe respiratory complications [51,52], a stunning of the phrenic nerve is speculative.

### Cephalic and extracephalic tDCS reference electrode

The modern safety guidelines for tDCS [53] recommend using bi-cephalic montages, which have the obvious advantage of avoiding any stimulation of the brainstem but introduce an ambiguity: are the observed effects exclusively due to modulation of the target cortical area activity or to the combination of the modulation of the target area and of the contralateral cortex located under the so-called "reference" electrode? It is only recently that this issue was partly resolved by using a large "reference" electrode placed over the contralateral orbita [54]. Since this large electrode is theoretically neutral (the current under the large electrode is so dispersed that it should be ineffective), the effects observed could be attributed solely to the modulation of the target M1 by a small "active" electrode.

Nevertheless, using an extracephalic reference electrode could lead to two opposite but equally interesting conclusions. On the one hand, if the direction of the DC flow is a key factor determining the (after-)effects of tDCS as suggested by recent modelling studies [7-13], then using an extracephalic reference electrode may potentially expand the variety of potential interventions with tDCS.

On the other hand, the impact of tDCS on a given cortical target may be insensitive to the DC flow direction, whether a cephalic or an extracephalic reference electrode is used. Therefore, using an extracephalic electrode as a genuine neutral reference would help to substantiate the conclusions of several recently published tDCS studies with bi-cephalic montages regarding the spatial location of the observed tDCS effects. Whereas the measures of the cortical excitability of M1 after tDCS are not influenced by a "reference" electrode placed over the controlateral frontopolar cortex, the issue is much more debatable for cognitive studies or studies using behavioural outcome measure. As long as behavioural studies are concerned, the definition of a cortical area as "functionally inert for the relevant task" for a control experiment may be questioned. These control experiments are of course of paramount importance but their interpretation may not be as straightforward as commonly accepted.

### Limitations of the study

Several issues should be taken in account when evaluating the outcomes of the present experiment. Firstly, whereas bi-frontal electrodes were used by Lippold and collaborators [14,15], in this experiment, a single cephalic electrode was placed on Fz in order to maximise the chance of directing the DC flow towards the brainstem. Moreover, during pilot experiences, positioning the cephalic electrode more anteriorly (i.e. over FPz) induced a typical metallic taste in the mouth in most subjects, which raised concerns for the double-blind character of the experiment.

Secondly, as already mentioned, the intensity of the DC was much larger in the study of Lippold et al [14,15] (16 minutes of 3 mA bi-frontal cathodal tDCS); this may explain the lack of effect observed in the present experiment. However, we deliberately decided to apply 20 minutes of 1 mA tDCS because 1) these parameters were used in the majority of the modern tDCS studies, 2) the blinding of healthy subjects is questionable when using high tDCS intensities such as 3 mA (tingling and itching cutaneous sensations), and 3) this study was designed as a first step towards other experiments exploring different parameters (larger cohorts of healthy volunteers, higher tDCS intensities, different locations of the cephalic electrode, inclusion of patients).

## Conclusions

Although this comprehensive study expands and confirms recent reports suggesting a lack of interference of tDCS with an extracephalic reference electrode with vital functions [20-23,34,35], it has several limitations. First, as discussed previously, only a midline frontal - right lower limb montage was explored, leaving unanswered the question whether tDCS applied on lateral aspect of the brain (particularly over the insular and parietal cortices) could modulate vital parameters or autonomic nervous functions. Second, since only healthy volunteers were included in this exploratory study, the conclusions may not apply to patients with brain or cardiovascular diseases. Third, whereas 20 minutes of tDCS at 1 mA has been used in most of the physiological studies, higher intensities, longer durations and repeated intervention should be tested in order to establish the safety of this montage.

A conservative interpretation of the present results would suggest that, using the same electrode montage and tDCS parameters, interference of tDCS with an extracephalic reference electrode at the level of the brainstem should be rather limited if any, and can be considered as safe in healthy volunteers. Further experiments are warranted to verify whether this conclusion may be extended to other tDCS parameters (electrodes montage, intensity, duration, etc) and to patients. Therefore, the use of an extra-cephalic reference electrode in future tDCS studies would expand the field of tDCS experiments by allowing to test different DC flow direction or help to confirm the conclusions of several modern tDCS studies regarding the spatial location and the real impact of concomitant bi-polar stimulation of different parts of the brain as inherently provided by bi-cephalic tDCS montages. Since tDCS have several features that place it in the pole-position to succeed in the translation from bench to bedside for therapeutic use in the real world, the safety aspect and potentially differential physiological effect of tDCS with an extracephalic electrode requires further investigations.

## Abbreviations

ANOVA_RM_: (repeated measures analysis of variance); BMI: (body mass index); dBP: (diastolic blood pressure); DC: (direct current); GEE: (generalised estimating equations); HF-band: (high frequency band); HF nu-RRI: (vagal tone of RRI in normalised unit: (nu), %); HR: (heart rate); HRV: (heart rate variability); LF-band: (low frequency band); LF nu-RRI: (sympathetic tone of RRI in normalised unit: (nu), %); LF/HF-RRI: (sympathovagal balance of RRI); M1: (primary motor cortex); PSD-RRI: (Power Spectral Density of RRI, ms^2^); RF: (respiratory frequency); RSA: (respiratory sinus arrhythmia); rTMS: (transcranial magnetic stimulation); RRI: (R-R interval); sBP: (systolic blood pressure); SD: (standard deviation); tDCS: (transcranial direct current stimulation).

## Authors' contributions

YV co-conceived of the study, participated in its design; participated in data acquisition, processing and interpretation; and drafted the manuscript. JJ participated in the design of the study, performed the statistical analysis and helped to draft the manuscript. MO co-conceived of the study, participated in its coordination; participated in data acquisition, processing and interpretation; and helped to draft the manuscript. All authors read and approved the final manuscript.

## Supplementary Material

Additional file 1 **Demographics and baseline data (mean ± SD)**. As can be appreciated from the Additional file, the three groups were balanced for age and sex; demographic and characteristics baseline heart rate, blood pressure are also provided for each subject. BMI: body mass index, RF: respiratory frequency, sBP(dBP): systolic(diastolic) blood pressure, HR: heart rate, LF(HF) nu-RRI: low (high) frequency band of RRI in normalised unit, PSD-RRI: Power Spectral Density of RRI, LF/HF RRI: LF/HF ratio of RRI. Click here for file
