# Supplementary material for: Effect of tDCS with an extracephalic reference electrode on cardio-respiratory and autonomic functions
Source: BMC Neurosci. 2010 Mar 16;11:38. doi: 10.1186/1471-2202-11-38 (PMC2844382; doi:10.1186/1471-2202-11-38)
Supplement: Additional file 1 — Demographics and baseline data (mean ± SD). As can be appreciated from the Additional file, the three groups were balanced for age and sex; demographic and characteristics baseline heart rate, blood pressure are also provided for each subject. BMI: body mass index, RF: respiratory frequency, sBP(dBP): systolic(diastolic) blood pressure, HR: heart rate, LF(HF) nu-RRI: low (high) frequency band of RRI in normalised unit, PSD-RRI: Power Spectral Density of RRI, LF/HF RRI: LF/HF ratio of RRI. [file 1471-2202-11-38-S1.DOC]

**Demographics and baseline data (mean ±** SD).

| **tDCS** | **subject** | **age (y)** | **sex** | **BMI** | **RF (Hz)** | **sBP (mmHg)** | **dBP (mmHg)** | **HR (bpm)** | **LF nu-RRI (%)** | **HF nu-RRI (%)** | **PSD-RRI (ms²)** | **LF/HF RRI** |
| --- | --- | --- | --- | --- | --- | --- | --- | --- | --- | --- | --- | --- |
| **sham** | 1 | 23 | f | 16,9 | 22,4 | 106,7 ± 3,5 | 65,5 ± 3,7 | 77,68 ± 5,19 | 74,48 ± 8,35 | 25,52 ± 8,35 | 337,87 ± 1.345,88 | 3,54 ± 2,80 |
|  | 2 | 36 | m | 21,46 | 20,7 | 132,6 ± 2,3 | 87,9 ± 2,3 | 70,53 ± 6,04 | 66,51 ± 7,29 | 33,49 ± 7,29 | 1.118,50 ± 848,86 | 2,23 ± 1,72 |
|  | 3 | 22 | m | 28,07 | 22,6 | 140,3 ± 3,6 | 87,3 ± 3,3 | 59,11 ± 5,07 | 45,52 ± 5,27 | 54,48 ± 5,27 | 3.528,68 ± 415,27 | 0,85 ± 0,18 |
|  | 4 | 42 | m | 22,05 | 17,9 | 121,7 ± 5,5 | 81,1 ± 4,1 | 62,97 ± 5,95 | 88,61 ± 3,96 | 11,39 ± 3,96 | 1.462,62 ± 2.848,20 | 11,03 ± 28,56 |
|  | 5 | 37 | f | 23,88 | 20,5 | 108,3 ± 4,3 | 72,4 ± 2,6 | 67,73 ± 3,31 | 71,04 ± 5,84 | 28,96 ± 5,84 | 690,62 ± 223,47 | 2,64 ± 1,43 |
|  | 6 | 35 | f | 23,44 | 21,5 | 108,4 ± 6 | 70,4 ± 4,8 | 68,60 ± 4,64 | 72,26 ± 7,51 | 27,74 ± 7,51 | 1.155,90 ± 667,69 | 2,95 ± 1,43 |
|  | 7 | 43 | m | 24,98 | 15,1 | 110,7 ± 4,3 | 75,6 ± 3,6 | 48,10 ± 9,88 | 56,24 ± 7,56 | 43,76 ± 7,56 | 12.111,75 ± 4.377,65 | 1,39 ± 1,05 |
|  | 8 | 30 | f | 21,11 | 19,8 | 103,9 ± 5,1 | 61,3 ± 4,5 | 63,26 ± 6,87 | 23,91 ± 8,04 | 76,09 ± 8,04 | 4.437,76 ± 1.064,84 | 0,33 ± 0,17 |
|  | 9 | 24 | f | 20,28 | 14,8 | 110,8 ± 13,9 | 72,3 ± 16,2 | 56,76 ± 8,59 | 63,54 ± 7,28 | 36,46 ± 7,28 | 4.402,20 ± 2.456,74 | 1,84 ± 0,52 |
|  | 10 | 47 | m | 23,67 | 15,2 | 114 ± 3,9 | 75,6 ± 3,5 | 69,31 ± 7,31 | 36,16 ± 9,96 | 63,84 ± 9,96 | 1.627,95 ± 358,42 | 0,62 ± 0,34 |
|  | **mean ± SD** | **33,9 ± 8,9** | **-** | **22,58 ± 3** | **19 ± 3** | **115,7 ± 12** | **75 ± 8,6** | **64,41 ± 8,31** | **59,83 ± 19,56** | **40,17 ± 19,58** | **3087,38 ± 3509,79** | **2,74 ± 3,09** |
| **cathodal** | 11 | 27 | f | 22,66 | 20,7 | 115,5 ± 4,9 | 72,5 ± 5 | 63,50 ± 7,04 | 36,20 ± 8,15 | 63,80 ± 8,15 | 2.916,01 ± 656,43 | 0,59 ± 0,21 |
|  | 12 | 32 | f | 20,31 | 21,9 | 107,7 ± 3,8 | 72,3 ± 3,2 | 71,38 ± 4,04 | 73,88 ± 9,35 | 26,12 ± 9,35 | 701,69 ± 255,59 | 3,41 ± 2,29 |
|  | 13 | 39 | m | 24,86 | 17,9 | 119,1 ± 3,5 | 79,2 ± 3,4 | 62,38 ± 6,36 | 51,33 ± 9,84 | 48,67 ± 9,84 | 1.033,40 ± 521,59 | 1,14 ± 0,46 |
|  | 14 | 49 | f | 22,03 | 21,9 | 103,34 ± 4,1 | 66,1 ± 3,8 | 53,94 ± 6,87 | 36,99 ± 6,27 | 63,01 ± 6,27 | 4.776,78 ± 1.248,40 | 0,61 ± 0,19 |
|  | 15 | 36 | m | 22,68 | 18,4 | 126,5 ± 3,4 | 76,5 ± 2,9 | 73,02 ± 6,41 | 71,68 ± 8,83 | 28,32 ± 8,83 | 877,07 ± 1.005,13 | 3,13 ± 3,57 |
|  | 16 | 24 | f | 19,92 | 18,7 | 101,6 ± 3,8 | 63,2 ± 2,2 | 65,47 ± 3,64 | 30,70 ± 7,17 | 69,30 ± 7,17 | 565,49 ± 71,52 | 0,46 ± 0,17 |
|  | 17 | 40 | m | 29,01 | 22,5 | 126,2 ± 5,2 | 87,4 ± 3,6 | 67,43 ± 9,85 | 56,11 ± 5,99 | 43,89 ± 5,99 | 2.497,25 ± 1.382,68 | 1,33 ± 0,41 |
|  | 18 | 32 | m | 24,07 | 14,4 | 132,5 ± 6,8 | 79,4 ± 7,7 | 62,76 ± 8,08 | 75,31 ± 5,81 | 24,69 ± 5,81 | 4.651,62 ± 1.281,86 | 3,31 ± 1,17 |
|  | 19 | 37 | m | 21,39 | 17,3 | 131,6 ± 5,3 | 77,4 ± 3,9 | 72,42 ± 7,11 | 52,60 ± 9,87 | 47,40 ± 9,87 | 1.870,02 ± 708,80 | 1,24 ± 0,84 |
|  | 20 | 28 | f | 21,19 | 18,2 | 108,71 ± 4,1 | 70,9 ± 4 | 88,39 ± 7,69 | 50,95 ± 17,88 | 49,05 ± 17,88 | 488,96 ± 257,51 | 1,46 ± 1,41 |
|  | **mean ± SD** | **34,4 ± 7,4** | **-** | **22,8 ± 2,7** | **19,2 ± 2,6** | **117,3 ± 11,6** | **74,5 ± 7** | **68,07 ± 9,16** | **53,57 ± 16,10** | **46,43 ± 16,10** | **2037,83 ± 1634,78** | **1,67 ± 1,17** |
| **anodal** | 21 | 27 | m | 25,13 | 19,2 | 129,1 ± 4 | 79,5 ± 3,6 | 61,14 ± 4,85 | 68,80 ± 5,23 | 31,20 ± 5,23 | 2.349,46 ± 603,24 | 2,31 ± 0,86 |
|  | 22 | 26 | f | 17,67 | 18,4 | 108,1 ± 5,8 | 72,2 ± 4,4 | 58,24 ± 5,54 | 63,34 ± 10,94 | 36,66 ± 10,94 | 3.163,98 ± 1.782,49 | 2,05 ± 1,57 |
|  | 23 | 21 | f | 19,38 | 17,9 | 119,5 ± 5,5 | 78,9 ± 4,6 | 66,07 ± 6,66 | 27,60 ± 7,17 | 72,40 ± 7,17 | 4.041,21 ± 1.194,94 | 0,40 ± 0,15 |
|  | 24 | 26 | m | 20,6 | 17,6 | 121,5 ± 3,9 | 76,9 ± 3 | 75,36 ± 6,20 | 69,25 ± 11,73 | 30,75 ± 11,73 | 873,95 ± 1.455,69 | 3,19 ± 8,87 |
|  | 25 | 36 | m | 21,3 | 19,8 | 119,1 ± 5 | 73,5 ± 4,7 | 67,62 ± 7,33 | 79,54 ± 5,95 | 20,46 ± 5,95 | 1.599,23 ± 1.291,00 | 4,57 ± 7,07 |
|  | 26 | 44 | m | 23,15 | 19,2 | 128,5 ± 4,2 | 79,6 ± 4,8 | 63,37 ± 3,81 | 62,78 ± 7,66 | 37,22 ± 7,66 | 1.173,71 ± 481,82 | 1,84 ± 0,83 |
|  | 27 | 31 | f | 21,3 | 20,2 | 119,8 ± 5,5 | 76,6 ± 4,1 | 86,19 ± 6,01 | 70,83 ± 9,24 | 29,17 ± 9,24 | 429,74 ± 161,67 | 2,81 ± 1,35 |
|  | 28 | 31 | f | 22,53 | 18,4 | 107,6 ± 4,2 | 64, ± 4,63 | 52,72 ± 8,98 | 37,42 ± 5,33 | 62,58 ± 5,33 | 4.090,75 ± 1.101,72 | 0,61 ± 0,14 |
|  | 29 | 56 | f | 24,8 | 20,2 | 91,6 ± 11,1 | 57,4 ± 13,6 | 85,78 ± 4,86 | 68,46 ± 10,95 | 31,54 ± 10,95 | 48,99 ± 227,67 | 2,73 ± 2,07 |
|  | 30 | 33 | m | 33,87 | 20,9 | 139,5 ± 4,4 | 86,5 ± 4,6 | 87,00 ± 4,95 | 74,08 ± 10,75 | 25,92 ± 10,75 | 574,14 ± 2.682,73 | 3,69 ± 2,62 |
|  | **mean ± SD** | **33,1 ± 10,2** | **-** | **22,97 ± 4,46** | **19,3 ± 1,1** | **118,4 ± 13,4** | **74,5 ± 8,4** | **70,35 ± 12,50** | **62,21 ± 16,53** | **37,79 ± 16,54** | **1834,52 ± 1496,57** | **2,42 ± 1,29** |

As can be appreciated from the Additional file, the three groups were balanced for age and sex; demographic and characteristics baseline heart rate, blood pressure are also provided for each subject. BMI: body mass index, RF: respiratory frequency, sBP(dBP): systolic(diastolic) blood pressure, HR: heart rate, LF(HF) nu-RRI : low (high) frequency band of RRI in normalised unit, PSD-RRI :Power Spectral Density of RRI, LF/HF RRI : LF/HF ratio of RRI.
